# Type 2 diabetes markers in indigenous Argentinean children living at different altitudes

**DOI:** 10.3934/publichealth.2018.4.440

**Published:** 2018-11-16

**Authors:** Valeria Hirschler, Gustavo Maccallini, Claudia Molinari, Mariana Hidalgo, Patricia Intersimone, Claudio Gonzalez

**Affiliations:** 1 University of Buenos Aires, Argentina.; 2 Hidalgo Laboratories, Buenos Aires, Argentina; 3 University of Tucuman, Argentina

**Keywords:** type 2 diabetes risk, high altitude, indigenous children

## Abstract

**Background:**

Exposure to hypoxia at high altitude is increasingly being recognized as a risk factor for metabolic diseases.

**Objective:**

To determine the association between Type 2 diabetes (T2D) risk factors and altitude in two groups of Argentinean indigenous schoolchildren who live permanently at different altitudes.

**Methods:**

This cross-sectional study compared 142 schoolchildren from San Antonio de los Cobres (SAC), 3750 m above sea level, with 171 from Chicoana (CH), 1400 m. Data for children's anthropometry, blood pressure and lipids, as well as mothers' height and weight were assessed.

**Results:**

There was not a significant difference in age between SAC (9.0 + 2y) and CH (9.4 + 2y) children. However, mean children's weight (29 vs. 38 kg), height (130 vs. 138 cm), BMI (17 vs. 19 kg/m^2^), and HDL-C (46 vs. 48 mg/dL) were significantly lower in SAC than in CH, respectively. In contrast, systolic blood pressure (87 vs. 70 mmHg), cholesterol (157 vs. 148 mg/dL), and triglycerides (104 vs. 88 mg/dL) were significantly higher in SAC than in CH, respectively. There was not a significant difference in age (33.2 + 7y vs. 34.4 + 8y) and BMI (26.2 + 4y vs. 28 + 5y) between SAC and CH mothers. Multiple linear regression analyses showed that children's blood pressure (R^2^ = 0.38), triglycerides (R^2^ = 0.21), and HDL-C (R^2^ = 0.16) were significantly associated with altitude, adjusted for confounding variables.

**Conclusion:**

This study shows that indigenous Argentinean children living at 3750 meters have higher T2D risk compared with those living at 1400 meters above sea level.

## 1. Introduction

Cardiovascular disease (CVD) is the leading cause of death worldwide, with a greater incidence in developing countries. [1]. It has been suggested that the rates of metabolic diseases are lower among individuals living at high altitude [2]. However, a previous study showed that high-altitude dwellers may have increased risk for type 2 diabetes (T2D) [3] compared with their counterparts living at sea level. Approximately 7% of the world's population (∼440 million people) resides at an altitude greater than 1500 m [4]. Usual residence at an altitude of 2500 m or above is the conventional demarcation for high altitude [5]. The size of the population currently living above 2500 m is estimated to be over 140 million worldwide [5]. At such altitudes, some adaptive changes occur with time that affects different organs, systems, and overall metabolism. Both the barometric pressure and the absolute concentration of oxygen decline as a function of elevation [5]. Some factors have been proposed to explain this difference, such as interaction between biological and environmental factors (hypoxia, temperature, sunlight exposure), ethnicity, and socioeconomic status, among others [3],[5]. Furthermore, high-altitude research found a significant association between chronic hypoxemia and cardiometabolic risk in Andean indigenous population [6]. In Tibet, Sherpas living at high altitudes have a metabolic adaptation and a genetic selection associated with improved mitochondrial coupling and a possible compensatory increase in fatty acid oxidation protecting them from oxidative stress [7]. The excessive erythrocytosis, the hallmark of chronic mountain sickness, was associated with metabolic syndrome in the Andean indigenous population [6]. In addition, a previous study performed by our group found that indigenous Andean Argentinean children living at 3750 m above sea level had higher T2D markers than children from a mixed population in an urban setting, probably due to ambient hypoxia or ethnicity [8]. However, there is a lack of information about age-specific T2D markers in Argentinean indigenous children from similar ethnic backgrounds but living at different altitudes. Our group performed a cross-sectional study to compare the prevalence of T2D markers among indigenous children in San Antonio de los Cobres (SAC), at 3750 m above sea level, with that of children with a similar ethnic background from Chicoana (CH) living at 1400 m above sea level. The relevance of this study, which compares two groups of schoolchildren of similar ethnic backgrounds, is that it demonstrates the association between altitude and T2D markers, independent of ethnic origin. The objective of the study was to determine the association between T2D risk factors and altitude in two groups of Argentinean indigenous school children who live permanently at different altitudes.

## 2. Methods

### 2.1 Study design

A cross-sectional study was designed to compare T2D markers between two indigenous communities living at different altitudes. The study was approved by the Human Rights Committee. Each parent gave written informed consent after an explanation of the study and before its initiation.

The Andean region of South America was initially inhabited around 7000 BC. The different ethnic groups that lived in this region were indigenous Quechuas, Aymaras, Calchaquies, Diaguitas, and Huarpes. The word *Diaguita* was a name given by the Aymaras based on the Aymara word *thiakita*, which means ‘far away’ or ‘foreign’. The Diaguita people were defined as a single community, given their linguistic and racial aspects, as well as their socioeconomic organization [9]. They inhabited the hills and valleys of northwest Argentina, part of which is now the province of Salta. This study included two indigenous communities of Diaguita descent in the Salta province: SAC and CH.

The details of the SAC community have been reported previously [8]. However, a brief description is included in this article. SAC is a town located in the Andes Mountains, 3750 m above sea level with a population of 4274 inhabitants [9]. Ninety-eight percent of the SAC population is indigenous Koyas, who are descendants of the Diaguitas [9]. Because none of the parents had any history of intermarriage with other ethnic groups in their family trees, the group was ethnically homogenous. CH is a town located in the southwest of the Lerma Valley, 1432 m above sea level, with a population of 4202 inhabitants [9]. The population of CH is indigenous and of Diaguita descent. Even though CH is situated 170 km from SAC and very close to Salta, the capital of the province, the inhabitants of CH have maintained their ethnic homogeneity. Similar to the SAC children, none of the CH children who participated in this study had any history of intermarriage with other ethnic groups in their family trees.

**Figure 1.**
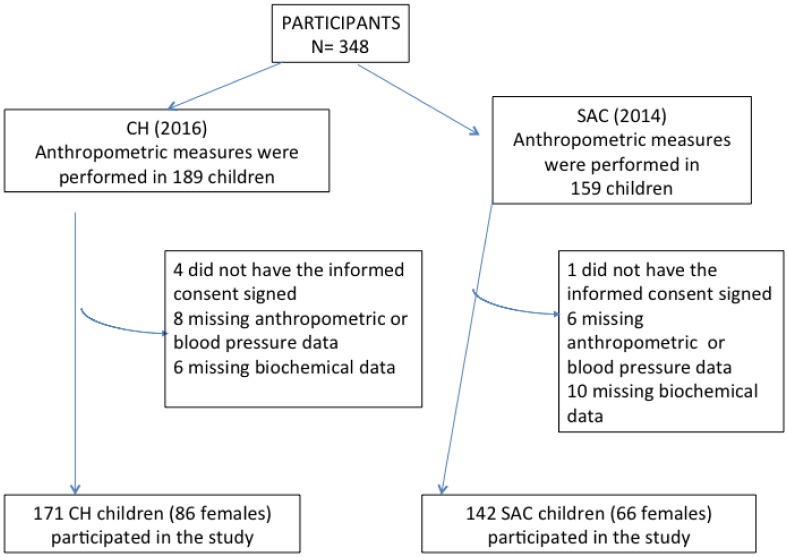
Study sample recruitment flowchart

One out of the three schools existing in SAC was selected by simple randomization in November 2014. Another school of comparable socioeconomic status was selected among the elementary schools located in CH in November 2016. The overall individual response rate was 90% (Figure 1). Assuming that the prevalence of hypertriglyceridemia was close to 45% in SAC and close to 30% in CH children, the sample size of 142 SAC and of 171 CH children would give a power of 0.80 at a significance level of 0.05.

Five sets of data were collected: demographic data, dietary intake, anthropometric measurements, blood pressure, and biochemical data. Healthcare professionals performed all measurements. Socio-demographic characteristics recorded included level of education and the presence or absence of a refrigerator or a dirt floor in the house. These two indicators are used to identify families of very low socioeconomic level by the National Statistics and Censuses Institute of Argentina [9].

Exclusion criteria included (1) missing anthropometric, blood pressure, or biochemical data, (2) the use of medication that would affect blood pressure, lipids, and glucose levels, and (3) the informed consent form not being signed. Participants included in our study sample had no significant difference in socioeconomic status, age, BMI, and waist circumference, with those that were excluded because of missing data. We chose to study mothers and not both parents as only mothers could attend the evaluation with their children. Most of the fathers worked full time and could not attend.

Height and weight were assessed with subjects wearing light clothing and without shoes. Height was recorded to the nearest 0.1 cm with a wall-mounted stadiometer. Weight was assessed to the nearest 0.1 kg on a medical balance scale. Body mass index (BMI) was calculated as weight in kilograms divided by height in meters squared.

Blood pressure was recorded with the child in the seated position by use of a mercury sphygmomanometer, with the child's right forearm horizontal on a table. Two readings were recorded at an interval of 1 to 2 minutes, and the cuff was completely deflated between the readings. The mean of the 2 readings was calculated for final analysis. Blood pressure percentiles were determined using the National Heart Lung and Blood Institute guidelines and adjusted for age, sex, and height percentile [10].

BMI z-score (BMI-z) and percentiles were also determined according to Center for Disease Control (CDC) norms [11]. Children were classified as underweight (<5th percentile), normal weight (5th to <85th percentile), overweight (85th to <95th percentile), or obese (≥95th percentile) [11]. Hypertension was defined by systolic and/or diastolic blood pressure ≥95th percentile, according to age, sex, and height, respectively [10]. Mothers' overweight was defined as BMI ≥25 and <30 kg/m^2^ and obesity as BMI ≥30 kg/m^2^ according to the National Cholesterol Education Program's Adult Treatment Panel III [12].

All samples were analyzed in a single laboratory. We had stored SAC and CH serum samples at −70°C and both groups were assessed together. Lipids were analyzed by standardized methods using the Architect c 16000 instrument (Toshiba, Kanagawa, Japan) and dedicated reagents (Abbott Laboratories, Illinois, USA). Two monthly CV's were shown because for each test, two levels of internal quality control materials were used. Inter-assay coefficients of variation (CVs) were the following: cholesterol 0.62 and 0.95%; HDL-C 2.00 and 3.08%; and triglycerides 0.87 and 1.11% respectively. Abnormal lipid levels were defined according to the National Institute of Health's Expert Panel on Integrated Guidelines for Cardiovascular Health and Risk Reduction in Children and Adolescents.

## 2.2 Statistics

Descriptive statistics for raw variables are presented as mean ± standard deviation values. The χ^2^ test was used to compare proportions. When more than 20% of the cells had expected frequencies of < 5, a Fisher's exact test was used. When comparing two groups with normally distributed data, a Student's *t* test was performed. Variables with a skewed distribution were logarithmically transformed for analysis. After log transformation, the data were tested again to confirm the findings. Bonferroni's adjustment was carried out when many comparisons were performed.

The primary focus of the study was to compare T2D markers between two indigenous communities living at different altitudes. Multiple linear regression analyses were performed to assess the association between blood pressure and lipid levels as dependent variables, and age, sex, BMI, location (SAC vs. CH), parental education, and maternal age and BMI as independent variables. *P* values of <0.05 were considered statistically significant. Analyses were performed using the SPSS (Chicago, IL) statistical software package SPSS version 22.0.

## 3. Results

Figure 1 shows the study flowchart. One hundred forty-two (66 females) SAC children were compared with 171 (86 females) CH children. Seventy-three percent of SAC and 43% of CH parents had no high school education (p < 0.01) and 14% of SAC and 3% of CH families did not have a refrigerator at home (p < 0.01). Even though all participating families from both communities were in the low socioeconomic class, socioeconomic levels were significantly lower in SAC than in CH. However, it is interesting to note that there was not a significant difference in age and BMI between SAC and CH mothers (Table 1).

**Table 1.** Clinical characteristics.

	SAC (n = 142)	CH (n = 171)
Age in years at screening	9.0 ± 2.1	9.4 ± 2.1
Birth weight (kg)	3.1 ± 0.5	3.2 ± 0.6
Weight (kg)	29.3 ± 8.8	37.5 ± 13.7**
Waist circumference (cm)	60 ± 8	67 ± 13**
Height (cm)	130 ± 12	137 ± 13**
z-Height	−0.8 ± 0.8	0.75 ± 1.1**
BMI (kg/m^2^)	16.8 ± 2.7	19.3 ± 4.4**
Z-BMI	−0.1 ± 1.0	0.7 ± 1.2**
Maternal age in years	33 ± 7	34 ± 8
Maternal BMI (kg/m^2^)	26 ± 4	29 ± 5
Systolic BP (mm Hg)	87 ± 14	70 ± 14**
Diastolic BP (mm Hg)	58 ± 14	48 ± 11**
Mean arterial BP (mm Hg)	67 ± 13	55 ± 14**

BMI: body mass index; BP: blood pressure. Data are presented as mean ± SD. Z-score is a quantitative measure of the deviation of a specific variable taken from the mean of that population. CDC z-BMI takes into account age and sex. Significance: *p < 0.05 and **p < 0.01.

### 3.1 Clinical and Metabolic Characteristics in SAC and CH children

Table 1 describes clinical characteristics of the sample. There was not a significant difference in age and birth weight between SAC and CH children. However, mean weight, height, and BMI, adjusted for age and sex, were significantly lower in SAC than in CH children. In contrast, systolic, diastolic, and mean blood pressures were significantly higher in SAC than in CH children.

Table 2 describes metabolic characteristics of the sample. Glucose levels were significantly higher in SAC than in CH children. Regarding the lipoprotein profile, HDL-C levels were significantly lower in SAC than in CH children, whereas total cholesterol, non HDL-C, and triglycerides were significantly higher in SAC than in CH. Furthermore, different lipid-related indexes were calculated. Interestingly, total cholesterol/HDL-C, LDL-C/HDL-C, and triglycerides/HDL-C ratios were significantly higher in SAC than in CH children. However, insulin levels were significantly lower in SAC than in CH.

**Table 2.** Metabolic characteristics.

	SAC (n = 142)	CH (n = 171)
Cholesterol (mg/dL)	157 ± 25	148 ± 35**
HDL-C (mg/dL)	46 ± 8	48 ± 11#
LDL-C (mg/dL)	90 ± 22	83 ± 22**
Triglycerides (mg/dL)	104 ± 39	88 ± 41**
Triglycerides/HDL-C	2.4 ± 1.1	1.9 ± 1.2**
LDL-C/HDL-C	2.0 ± 0.6	1.8 ± 0.5**
Cholesterol/HDL-C	3.5 ± 0.7	3.1 ± 0.8**
Non HDL-C (mg/dL)	111 ± 25	100 ± 32**
Glucose (mg/dL)	90 ± 7	80 ± 6**
Insulin (IU/dL)	5.3 ± 2.8	6.9 ± 7.9**

HDL-C: high density lipoprotein cholesterol; LDL-C: low density lipoprotein cholesterol. Data are presented as mean ± SD. Significance: **p < 0.01, #p < 0.01 Mann Whitney test.

### 3.2 Prevalence of T2D risk factors

**Figure 2.**
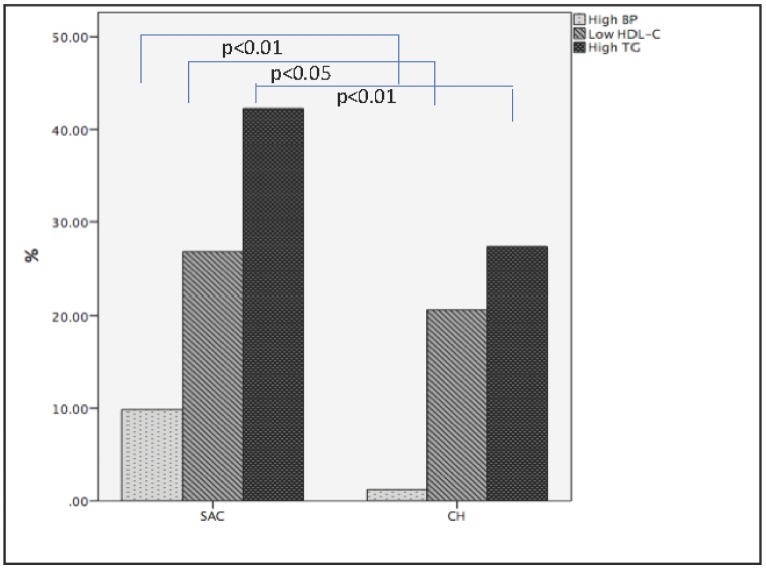
Prevalence of hypertension, low HDL-C, and high triglycerides (TG) in SAC & CH children. The x-axis represents SAC and CH children and the y-axis represents the percentage of T2D markers. SAC children had a higher prevalence of hypertension, low HDL-C, and high TG than CH children. Significance: p < 0.05

The prevalence of children's overweight/obesity was significantly lower in SAC 9.2% (n=13) than in CH 41.5% (n=71) children. There was not a significant difference in age and sex between normal weight and overweight/obesity in SAC and CH children. Figure 2 describes the prevalence of T2D markers in SAC and CH children. The prevalence of hypertension was significantly higher in SAC 9.9% (n=14) than in CH 1.1% (n=2) children. Therefore, the prevalence of hypertension was approximately ten-fold higher in SAC than in CH children, whereas the prevalence of overweight and obesity was approximately four-fold lower in SAC than in CH children. None of the children had abnormal glucose levels. Furthermore, the prevalence of hypertriglyceridemia was significantly higher in SAC 42.3% (n=60) than in CH 27.5% (n=47) children. Even though the prevalence of low HDL-C was higher in SAC 26.8% (n=38) than in CH 20.5% (n=35), the difference did not reach significant levels. There was not a significant difference in the prevalence of T2D markers between sexes in both communities.

### 3.3 Factors Associated with T2D Markers

Multiple linear regression analyses showed that children's mean blood pressure was significantly associated with altitude and children's age and BMI (R^2^ = 0.38); mean glucose levels were significantly associated with altitude and children's age and sex (R^2^ = 0.43); triglyceride levels were significantly associated with altitude and children's BMI (R^2^ = 0.21); and HDL-C was significantly associated with altitude and children's BMI and age (R^2^ = 0.16), adjusted for age, sex, parental education, and maternal age and BMI (Table 3). Furthermore, multiple logistic regression analyses showed that SAC children had thirteen-fold the odds of having hypertension (OR 13.3; 95% CI 2.7–64.8), and three fold the odds of having high triglycerides (OR 3.4; 95% CI 1.7–6.0) compared with CH children, adjusted for age, sex, and BMI.

**Table 3.** Multiple linear regression analyses

Dependent variables	Independent variables	B (standardized coefficient)	R^2^	P value
Glucose				
	Sex	−0.30	0.43	<0.01
	Age	0.12		0.20
	BMI	0.04		0.29
	Location	−0.55		<0.01
	Parental education	−0.02		0.73
	Mothers' BMI	0.02		0.84
	Mothers' age	−0.08		0.25
Mean BP				
	Sex	0.07	0.38	0.31
	Age	0.14		0.03
	BMI	0.14		0.04
	Location	−0.62		<0.01
	Parental education	0.06		0.34
	Mothers' BMI	0.02		0.72
	Mothers' age	−0.02		0.70
Triglycerides				
	Sex	−0.08	0.21	0.30
	Age	−0.01		0.95
	BMI	0.42		<0.01
	Location	−0.30		<0.01
	Parental education	−0.05		0.48
	Mothers' BMI	−0.14		0.06
	Mothers' age	0.02		0.78
HDL-C				
	Sex	0.03	0.16	0.63
	Age	0.26		<0.01
	BMI	−0.29		<0.01
	Location	0.26		<0.01
	Parental education	0.07		0.32
	Mothers' BMI	0.05		0.49
	Mothers' age	−0.03		0.72

BMI: body mass index; BP: blood pressure. Separate multiple linear regression models, adjusted for age, sex, children's BMI, location (CH vs. SAC), mothers' education, BMI, and age. Dependent variables: BP, glucose, triglycerides and HDL-C.

## 4. Discussion

This cross-sectional study shows that indigenous children from a similar ethnic background had increased levels of T2D markers when they lived permanently at higher altitudes than those living at lower altitudes. Furthermore, SAC children (3750 m above sea level) had significantly higher arterial blood pressure, glucose, and triglycerides, whereas HDL-C levels were lower than CH children (1400 m above sea level) adjusted for confounding variables. However, the prevalence of overweight/obesity was significantly lower in SAC than in CH indigenous children. Consistently, a previous study performed by our group involving indigenous SAC children showed a higher prevalence of dyslipidemia with a lower prevalence of obesity than urban children from a mixed population in Buenos Aires at sea level [8]. As far as we know, this is the first study in Argentina to examine the effect of altitude on T2D markers in two similar indigenous communities living at different altitudes. This study shows a higher prevalence of dyslipidemia and hypertension in SAC children compared with a community of a similar background but living at lower altitudes.

The prevalence of overweight/obesity was significantly lower in SAC than in CH indigenous children. Consistently, a cross-sectional study conducted at different altitudes, from 1200 to 3700 meters above sea-level, in the Everest region of Nepal showed that obesity decreased with an increasing level of altitude [13]. There are several plausible mechanisms relating to elevation and obesity, including socioeconomic status, nutrition, hypoxia, mean annual temperatures, physical activity, leptin signaling, and metabolic demands. It has been suggested that reduced temperature at increased elevation may lead to weight loss through catabolic effects [13]. Metabolic expenditures are required to cope with extreme temperatures [14]. Consistently, SAC is located in the mountains at 3750 m above sea level with a mean annual temperature of 7.7°C, and a mean annual wind speed of 21 km/hour [9]. Therefore, despite a high level of sun exposure, SAC children must endure a cold and windy climate. This could be the reason for the low prevalence of obesity in SAC children. In contrast, even though both communities belong to a similar background, CH is located at 1400 m with a mean annual temperature of 17.2°C [9]. Another possible mechanism that may contribute to the lower prevalence of obesity in SAC could be the differences in energy expenditure in physical activity [13]. Because SAC is located in mountainous terrain, children in SAC may expend more energy than CH children because they need to walk uphill on a regular basis. In contrast, CH children may expend less energy even if they walk the same distance every day. Finally, exposure to hypoxia has been shown to stimulate hypoxia inducible factor 1, which appears to be an important regulator for the expression of the leptin gene—a hormone secreted by adipose tissue that produces negative feedback on appetite—and inversely associated with obesity [15]. Consistently, this study showed that the prevalence of overweight and obesity was approximately four-fold lower in SAC than in CH children. In summary, this finding presents a striking example of the variation in the prevalence of obesity found in populations of similar ethnic backgrounds in different environmental circumstances; suggesting that if SAC children were to move to a location at lower altitudes the different conditions could lead to a greater T2D risk.

In this study, we were able to take into account several potential markers for childhood hypertension, including family socioeconomic status and maternal BMI, which have been shown to be associated with hypertension [16]. A previous study showed an association between low socioeconomic class in early childhood and hypertension [17]. Also, data from a systematic review of 122,053 adolescents indicate that the prevalence of high blood pressure was higher among children from low- and middle-income countries [18]. Even though both communities belonged to a low socioeconomic class, SAC families were of significantly lower socioeconomic status. Because of the possible influence of parental BMI on childhood hypertension [3], the present study introduced maternal BMI in the regression analyses. The prevalence of hypertension in SAC children was nine-fold higher than in CH children. Similar findings were reported in children from Pakistan with a prevalence of hypertension of 12.2% [19]. A possible reason could be the differences in body stature; an equally high BMI confers higher prevalence of hypertension in Asian than in white populations [20]. Genetic differences might contribute to the observed differences between SAC and CH children, although these two communities share a similar ancestry. Therefore, this leads us to believe that altitude could be the main variable associated with the increase of blood pressure levels in SAC children [21]. In addition, chronic exposure to high altitude results in increased sympathetic and parasympathetic activities, which lead to raised blood pressure [21]. Furthermore, the reduction of partial pressure gradients makes gas exchange more difficult, resulting in a long-term chronic insufficiency in oxygenated blood circulation [22]. This causes a drop-in blood supply to the kidneys, and the subsequent release of renin that contributes to vasoconstriction of arteries [22]. It has been suggested that the applied method for measuring blood pressure could be affected by altitude. The measurement performed is a relative one, that is, independent from the atmospheric pressure and altitude [23]. Therefore, based on the technical aspects of oscillometric and mercury blood pressure monitors, there was no reason to assume that altitude and/or lower barometric pressure would have any effect on their accuracy [23].

It is interesting to note that the prevalence of dyslipidemia, another T2D marker, was significantly higher in SAC than in CH children. In contrast, the results of other studies have generated mixed results. A study performed in the virtually complete adult population of Switzerland showed a significant and inverse association between individuals living at higher altitude and the risk for T2D; adjusted for confounding variables [24]. They suggested that favorable changes associated with exposure to moderate altitude were associated with beneficial lipid metabolism [25]. However, this study was performed in participants that lived at moderate altitude (600–1500 m) in a developed country. In contrast, the higher altitudes (3750 m), the cold, poor nutrition, socioeconomic problems, and geographical isolation of the SAC community may also play an important role in the development of dyslipidemia [6],[8],[9]. Consistent with our results, a study performed in the San Pedro de Cajas district, located in the Central Andes of Peru at an altitude of 4,100 m, showed a high prevalence of hypertriglyceridemia (53.9%) and of low HDL (45.3%) in indigenous descendants of the Amerindian populations, suggesting a relationship between dyslipidemia and future T2D [26]. Furthermore, a previous study found a significant association between chronic hypoxemia and cardiometabolic risk in Andean indigenous populations [6]. In addition, a study performed by our group in SAC school children showed a higher prevalence of dyslipidemia compared with urban Buenos Aires children living at sea level [8]. Accordingly, the present study found that SAC children had higher triglyceride and lower HDL-C concentration than CH children with both groups belonging to a similar ethnicity; suggesting that altitude might be the main factor associated with dyslipidemia. Moreover, high atherogenic risk is also indicated by higher different lipid-related indexes such as total cholesterol/HDL-C, triglycerides/HDL-C, and LDL-C/HDL-C in the SAC community [27]. Furthermore, higher levels of triglycerides/HDL-C index were associated with increased proportion of small and dense LDL particles [28],[29]. It is interesting to note that the above-mentioned abnormalities in the lipoprotein profile were present even though the prevalence of obesity was lower in SAC than in CH children, thus highlighting the concurrence of other conditioning factors such as oxidative stress. It is likely that although obesity may be less prevalent at high altitude, individuals with a lower oxyhemoglobin saturation have a higher risk for T2D [30]. However, the cold weather, poor nutrition, socioeconomic problems, and geographical isolation may also play an important role [8],[26]. Exposure to high altitude has been associated with increased lipid peroxidation [31]–[33]. Hypoxia could inhibit oxidative phosphorylation and could stimulate the oxygen signaling pathway through hypoxia-inducible factor-1 [34]. Hypoxia-inducible factor under hypoxic conditions regulates the expression of several genes that mediate adaptive responses to low oxygen tension in different tissues, suggesting that high altitude populations, regardless of their ethnicity, may display a genetic adaptation to hypoxia via the hypoxia-inducible factor pathway [34]. It is likely that although obesity may be less prevalent at high altitude, participants with lower oxyhemoglobin saturation have a higher risk for T2D [31].

Limitations and Strengths: This study has several limitations that should be acknowledged. First, this cross-sectional study cannot imply a causal relationship. Second, this study lacked information regarding family history of T2D markers, pubertal status, energy expenditure, physical activity, and diet history, all of which are known to influence T2D. Third, the study of the CH sample was not concurrent with that of the SAC sample and climatic or socioeconomic differences may have affected the results. Finally, the results of this study, conducted in a sample of indigenous SAC and CH children, may not be directly extended to children of other indigenous groups without confirmation. Despite these limitations, this study contributes to the literature by examining two similar indigenous communities. Other strong points of this study are the lack of relevant ethnic differences between highlanders and lowlanders and the fact that the study population was distributed in two different altitudes, allowing us to examine the association independently of ethnicity. Furthermore, there was a high response rate of the children, and the data were collected through measurements taken by our team rather than self-reported in school children. Finally, we used regression models and simultaneous adjustment of confounding variables.

## 5. Conclusions

The results of our study suggest that indigenous children who live permanently at high altitude, 3750 m, have higher T2D markers independently of age, sex, BMI, ethnicity, parental education, and maternal BMI. However, the prevalence of overweight/obesity was significantly lower in SAC than in CH children. Therefore, we concluded that altitude might be the most significant factor associated with T2D markers because both communities were from similar ethnic backgrounds and lived at a difference of more than 2000 m in altitude. Future randomized longitudinal studies should be performed to confirm our findings.
